# Looking for the best strategy to treat children with new onset juvenile idiopathic arthritis: presentation of the “comparison of STep-up and step-down therapeutic strategies in childhood ARthritiS” (STARS) trial

**DOI:** 10.1186/s12969-022-00739-x

**Published:** 2022-09-07

**Authors:** Marco Burrone, Marta Mazzoni, Roberta Naddei, Angela Pistorio, Maddalena Spelta, Silvia Scala, Elisa Patrone, Marco Garrone, Maria Lombardi, Luca Villa, Giulia Pascale, Roberto Cavanna, Nicolino Ruperto, Angelo Ravelli, Alessandro Consolaro, for the Paediatric Rheumatology International Trials Organisation (PRINTO)

**Affiliations:** 1 IRCCS Istituto Giannina Gaslini, Clinica Pediatrica e Reumatologia, Pediatric Rheumatology Unit, Genoa, Italy; 2 IRCCS Istituto Giannina Gaslini, Direzione Scientifica, Genoa, Italy; 3 IRCCS Istituto Giannina Gaslini, UOSID Centro Trial, PRINTO, Genoa, Italy; 4 EULAR Centre of Excellence in Rheumatology 2008-2023, University of Genoa, Università degli Studi di Genova, Dipartimento di Neuroscienze, Riabilitazione, Oftalmologia, Genetica e Scienze Materno-Infantili (DiNOGMI), Via Gerolamo Gaslini 5, 16147 Genoa, Italy

**Keywords:** Juvenile idiopathic arthritis, Anti-TNF, Treat-to target, Randomised clinical trial, Methotrexate

## Abstract

**Background:**

Although a satisfactory disease control is nowadays achievable in most patients with JIA, a substantial proportion of them still do not respond adequately or reach long-term drug-free remission. According to current recommendations, treatment should be escalated in subsequent steps. A different approach is based on the assumption that the initial start of an aggressive therapy may take advantage of the “window of opportunity” and could alter the biology of the disease, leading to an improvement of long-term outcomes, including the prevention of cumulative joint damage.

**Objectives:**

This randomised clinical trial aims to compare the effectiveness of a conventional therapeutic regimen, based on treatment escalation and driven by the treat-to-target approach, with that of an early aggressive intervention based on the initial start of a combination of conventional and biological DMARDs.

**Methods:**

JIA patients with oligoarthritis or RF negative polyarthritis aged more than 2 years and with less than 4 months of disease course will be included in the study. Children will be randomised into two arms: patients in Step-up arm with less severe oligoarthritis will undergo an intra-articular corticosteroid injection (IACI) in all affected joints; patients with polyarthritis or severe oligoarthritis will receive IACI and methotrexate. Subsequent treatment will follow a standardised protocol based on the patients’ level of disease activity measured with the JADAS, according to a treat-to-target strategy. Patients in Step-down arm will receive a 6-month early combined treatment (methotrexate plus IACI for less severe oligoarthritis, methotrexate plus etanercept for severe oligoarthritis and polyarthritis). The primary endpoint is the frequency of achievement of the status of clinical remission (i.e. persistence of inactive disease for at least 6 months) at the 12-month visit. Safety events, physician-centred measures and parent/patient-reported outcomes will be collected through the Paediatric Rheumatology International Trials Organisation on line database.

**Expected results:**

The STARS trial aims to provide important evidence supporting the first-line treatment choices in the care of children with oligoarticular and polyarticular JIA. If the superiority of an early aggressive therapy will be demonstrated, this will demand further studies on the biological definition of the window of opportunity for JIA.

**Trial registration:**

The Trial is registered on the ClinicalTrials.gov registry (NCT03728478) on the 31st October 2018 and EU Clinical Trials Register on the 14th May 2018 (EudraCT Number: 2018–001931-27).

## Background

### Rationale

The “comparison of STep-up and step-down therapeutic strategies in childhood ARthritiS” (STARS) trial aims to investigate whether an early aggressive therapeutic intervention, based on the initial start of synthetic and biologic DMARDs (Step-down strategy), is superior to an approach based on treatment escalation conducted following the treat-to-target principle [1] (Step-up strategy), in children with juvenile idiopathic arthritis (JIA). The effectiveness of the two strategies will be assessed by comparing their ability to induce sustained clinical disease remission on/off treatment.

With a different approach, both interventions aim to obtain a quick and robust disease control and maintain it over time. Historically and according to the ACR recommendation [2], the treatment of JIA is escalated in subsequent steps. In general, treatment begins with NSAIDs or intra-articular joint injections, which, in case of insufficient effectiveness or disease flare, are replaced by or associated with a synthetic DMARD, such as methotrexate (MTX), sulphasalazine or leflunomide. In case of inadequate response and according to the current label indication, a biologic DMARD is usually prescribed in substitution of or in conjunction with the synthetic DMARD.

The treat-to-target strategy was first advocated for the treatment of adult rheumatoid arthritis in 2010, based on its successful use in chronic conditions such as hypertension and diabetes mellitus. This approach is based on the assumption that frequent adjustment of therapy intended to reach minimal levels of disease activity will lead to an improvement in patient outcomes according to quantitative indices [3, 4]. Key components of the treat-to-target include the explicit definition of a therapeutic target (such as clinical remission or low disease activity) at the beginning of the treatment and the verification of its achievement at each subsequent visit. If the target is reached the treatment is left unchanged, whereas if the target is not reached the treatment is intensified. The 2010 EULAR recommendations [5] for the management of rheumatoid arthritis were centered on the treat-to-target strategy, as were the later joint ACR/EULAR recommendations. A 2013 update of the EULAR recommendations [6] went even further in highlighting the treat-to-target as a fundamental component of treatment strategies. In 2018, the first recommendations for the application of the treat-to-target study to the care of children with JIA were developed. Although recommendations could not be based on high level of evidence, they reached significant agreement in an international task force of pediatric rheumatologists with a large experience in the field [4]. Evidence supporting the treat-to-target strategy is now increasing. The recent Best for Kids study [7], which aimed to compare three different treatment strategies for JIA (synthetic DMARD monotherapy, synthetic DMARD and bridging prednisone, and a combination of synthetic and biologic DMARDs), showed that regardless of initial specific treatments, after 24 months of treatment-to-target aimed at drug-free inactive disease, 71% of recent-onset patients with JIA had inactive disease and 39% were drug free. A non-randomised study showed that patients treated according to a treat-to-target protocol had a higher chance to achieve remission in comparison to a matched cohort [8]. A quality improvement initiative in a tertiary care medical centre showed that intense patient monitoring, target attestation and standardization of clinical decision support lead to better patient outcomes [9].

Early intensive therapy in JIA during the so-called window of opportunity is believed to be able to alter the biology of the disease and improve long-term outcomes, including prevention of cumulative joint damage [4, 10, 11]. In systemic JIA, early anti-IL-1 therapy was found to quickly lead to inactive disease and facilitate sustained disease control. Indeed, in a cohort of steroid-naive patients with new-onset systemic JIA treated with IL-1 receptor antagonist therapy, 85% of patients had achieved inactive disease or an ACR paediatric 90% response by 3 months of treatment. The benefits of early treatment with other agents and in other JIA categories are less clear, but have been increasingly suggested for polyarticular JIA. In the ACUTE-JIA trial [10], initiation of anti-TNF therapy plus MTX in DMARD-naive patients with polyarticular JIA was more effective in achieving minimally active or inactive disease than MTX alone or MTX plus hydroxy-chloroquine and sulfasalazine. The TREAT trial [11] showed a benefit with an early aggressive strategy, even though it failed to reach its primary end-point. More recently, a trajectory analysis of 400 patients in the Start Time Optimization of Biologics in Polyarticular JIA study demonstrated that starting a biologic treatment within 3 months of baseline assessment is associated with a more rapid achievement of inactive disease in subjects with untreated polyarthritis [12].

The approach proposed in the present trial represents a step forward as compared to most previous therapeutic studies in JIA, as it does not involve the exposure of children to a placebo. Furthermore, it does not assess the effectiveness of treatments in terms of mere percentage improvement in disease symptoms or achievement of a particular disease state at a single time point but it evaluates the ability of treatments to induce and maintain the state of clinical remission over time. In addition, the trial includes the administration of systemic corticosteroids only in the most severe disease subsets (i.e., those characterised by involvement of hip or cervical spine joints). Of note, a recent trial of early aggressive therapy in polyarticular JIA [11] has been subjected to major criticism owing to the choice of giving oral prednisone to all children in the more aggressive arms.

The design of the STARS trial is innovative and aims to address the major unmet needs in the current management of JIA in the real world of daily clinical care. For instance, it is still unclear whether progressive treatment intensification based on the magnitude of therapeutic response over time is more advantageous than initial administration of an aggressive therapy in terms of achievement and maintenance of complete disease control. Furthermore, it is still debated whether intra-articular corticosteroid therapy leads to better results with lower side effects than systemic corticosteroid administration. The Step-up approach will be conducted following the treat-to-target strategy, a novel method of management which has proved effective in the care of adult patients with rheumatoid arthritis and has shown better therapeutic results than conventional regimens in several controlled trials [13]. Note that recommendations for the use of the treat-to-target strategy in adults with rheumatoid arthritis have been recently issued by the European League Against Rheumatism (EULAR) [14]. Although there is an increasing interest among pediatric rheumatologists in the adoption of the treat-to-target strategy, this approach has not been hitherto implemented in the management of children with JIA.

In conclusion, the primary aim of the trial is to scrutinise the ability of the two therapeutic interventions to induce complete disease control early and to maintain it over time. Among other objectives, investigating their capacity to reduce the need for the later introduction of biologic agents, which would lead to a reduction in the costs for the national health-care system and in the exposure of children to the potential (and still unclear) long-term side effects of these medications. The achievement of the main endpoints of the trial might also result in the decreased need of repeated intra-articular corticosteroid joint injections (IACI), which might lessen the child’s and parents’ related emotional distress and the financial and organizational burden required by these procedures under sedation or general anaesthesia.

Specific aim of this work is to present the STARS trial to the international pediatric rheumatology community and to foster participation in the trial outside the Italian borders.

### Potential risks and benefits

Children in the STARS trial will be exposed only to synthetic and biologic disease modifying antirheumatic drugs (DMARDs) that had been licensed for use in JIA. Children in Step-up arm will be formally treated in accordance with current guidelines and drug licenses. Subjects in the Step-down arm will receive MTX (for less severe oligoarthritis patients) or etanercept (for polyarthritis and for more severe oligoarthritis patients) as a first-line treatment. Differently, in Europe, MTX is currently licensed only for use in polyarthritis and etanercept for polyarthritis when MTX has been demonstrated to be ineffective. However, around 70% of children with oligoarticular onset of JIA can expect to be prescribed MTX and around 50% of children with polyarthritis can expect to be prescribed etanercept throughout the course of their disease [15].

Treatment with MTX and etanercept is commonly believed to be associated with an increase of infection rate, in particular tuberculosis for etanercept. However, in a recent study with more than 8000 patients and 360,000 controls, the overall risk of acquiring an infection leading to hospital admittance was twofold higher in those with JIA than in those with attention deficit hyperactivity disorder [16]. This effect was already evident in patients with JIA not yet exposed to MTX or biologic agents and therefore is probably attributable to the immunological dysregulation associated with the chronic inflammatory disease. The risk of such infections was not further increased by use of MTX or etanercept. In contrast, treatment with high­dose glucocorticoids increased the infection risk by threefold. JIA itself confers risks for additional diseases, including autoimmunity and malignancies, especially lymphoma. In general, however, there is no direct evidence that an earlier introduction of these medications is associated with an increase of side effects frequency. In addition, MTX may a raise of liver enzymes; to minimise this risk, folinic acid is prescribed at one third to half the MTX dose the day after its administration. MTX can also cause severe nausea, thus anti-emetics are recommended as standard care. Other recommended measures in the trial to reduce risk from standard corticosteroid therapy include the use of proton pump inhibitors to minimise gastro-intestinal toxicity, and osteoporosis prophylaxis.

Treatment in both trial arms aims to reach a sustained and complete disease quiescence. The achievement of such state implies the disappearance of joint pain, morning stiffness and functional limitation. This objective may lead to restoration of the ability of the child to perform daily activities and to improve the quality of life of the child and the family. Sustained suppression of the inflammatory disease process may also help preventing long-term joint damage and, consequently, reduce the expenses of the health care system in terms of physiotherapy, need of devices (e.g. crutches, wheelchairs), orthopaedic surgery, etc. Another potential advantage of the therapeutic regimens assessed in the trial is avoiding disease exacerbations, which may require the prescription of systemic corticosteroids. Minimizing the use of these medications may lessen the frequency of serious adverse events secondary to their prolonged administration, particularly growth failure, weight gain, and cushingoid features. Sustained disease control may also reduce the need of repeated IACI, which cause distress to the child and the family and may increase the organizational and financial burden to the health care system in case general anaesthesia in the operatory theatre is needed. Broader objectives are the avoidance of absences of children from school and of parents from work, which may be caused by disease exacerbations or the request of frequent clinical visits or laboratory tests due to persistently active disease or continued treatment with potentially toxic medications. Particularly innovative aspects of the STARS trial include the use of standardised quantitative measures to assess the disease state and the disease course over time and the involvement of patients and parents in clinical decision making, through their assessment of disease activity by child-centred or patient-centred outcome measures.

## Methods

### Trial design

STARS is a 12-month multicentre randomised single-blinded superiority clinical trial of two different treatment strategies (Step-down versus Step-up) in a treat-to-target setting. After the conclusion of the observation period of the trial, patients will be followed for up to 5 years for the evaluation of the disease course, medication requirements, adverse events and long-term disease-related morbidity. Recruitment started on 21st May, 2019.

### Selection of centres

In a first phase, all Italian centres belonging to the Paediatric Rheumatology International Trials Organization (PRINTO) were invited to participate in the study. In January 2022, after a feasibility survey, PRINTO centers outside Italy were also invited to participate, based on the possibility to prescribe MTX in oligoarthritis and etanercept in polyarthritis as a first-line treatment according to local rules.

### Study population

Recent-onset JIA patients with oligoarthritis or rheumatoid factor negative polyarthritis aged between 2 and 17 will be included in the study. Participants must be DMARD-naïve (only treatment with one NSAID is allowed for no more than 6 weeks from diagnosis) and must not have received corticosteroid joint injections in the 3 months before enrolment.

After obtaining written consents (and assent where appropriate) from the participant, parent or legal guardian, patients’ disease severity will be assessed based on the clinical phenotype (irrespective of the ILAR classification) and the severity of the disease. Disease severity will be defined according to the extension of arthritis and based on the presence/absence of features of poor prognosis according to the 2011 American College of Rheumatology (ACR) recommendations for the treatment of JIA [2] (Table 1). Children with oligoarthritis without features of poor prognosis will be considered as “less severe patients”, children with RF-negative arthritis and children with oligoarthritis and features of poor prognosis will be considered as “more severe patients”. As stated in the 2011 recommendations, risk stratification is crucial for guiding optimal treatment; features of poor prognosis for oligoarthritis were defined by the recommendations panel based both on literature revision and on the panellists’ clinical experience [2]. In particular, the possible role of erythrocyte sedimentation rate as a predictor of a less favourable outcome for oligoarthritis patients was more recently confirmed by the results of a clinical trial on this subgroup of patients [17].

**Table 1:** Subjects in both arms are grouped according to the severity of the disease, based on the 2011 American College of Rheumatology recommendations for the treatment of juvenile idiopathic arthritis (ref. [2])

**More severe patients**	
- Children with RF negative polyarthritis	
- Children with oligoarthritis AND features of poor prognosis:	
a. Arthritis of the hip or cervical spine	
b. Arthritis of the ankle or wrist and marked (> 40 mm/h) or prolonged (> 20 in at least 3 consecutive assessments within 3 months) erythrocyte sedimentation rate elevation	
**Less severe patients**	
- Children with oligoarthritis WITHOUT features of poor prognosis	

### Interventions

After the screening visit and recording of informed consent, patients will be randomised into two treatment arms: “Step-up” and “Step-down”. Patients in the Step-up arm will be treated according to a conventional strategy based on treatment escalation and driven by the treat-to-target strategy. Patients in the Step-down arm will be treated with an early, combined, aggressive therapy for 6 months.

**Fig. 1:**
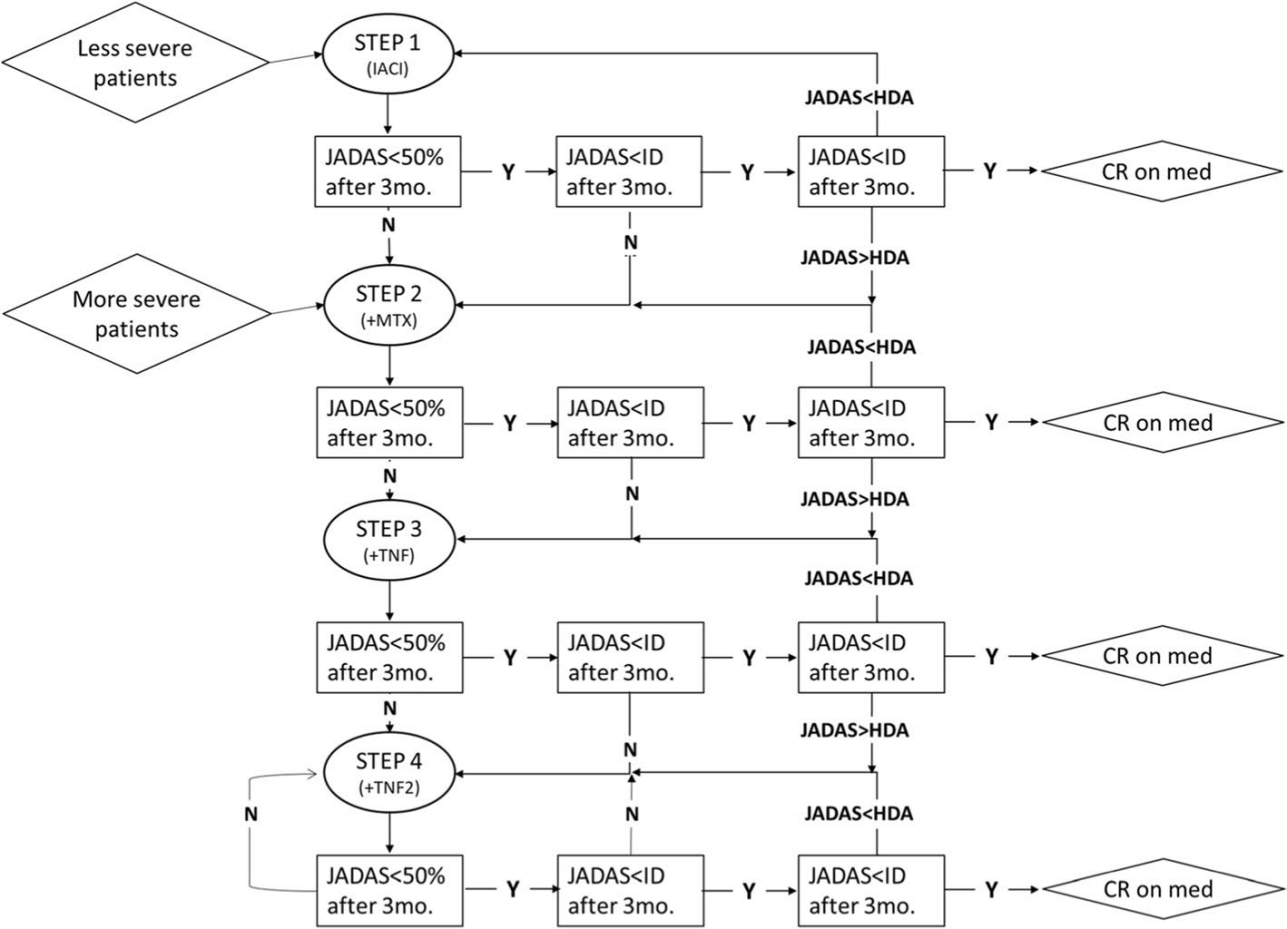
Step-up arm design. T2T Group 1: Subjects with oligoarthritis without features of poor prognosis. T2T Group 2: Subjects with oligoarthritis with features of poor prognosis and subjects with polyarthritis. JADAS: Juvenile Arthritis Disease Activity Score-10. JADAS< 50%: improvement of JADAS10 of at least 50%, JADAS<ID: JADAS10 score below inactive disease cutoff. JADAS<HDA: JADAS10 score under HDA cutoffs. JADAS>HDA: JADAS10 score above HDA cutoffs. If at the visit 3 months after step 1, 2, and 3 the patient is improved by less than 50% in the JADAS score, but he/she reaches the JADAS state of minimal disease activity, treatment is not escalated ID = inactive disease, HDA = high disease activity. CR on med.: clinical remission on medication. IACI: intra-articular corticosteroids injection. +MTX: start methotrexate (repeating IACI or adding a short course of prednisone can be considered). +TNF: add therapy with an anti-TNF agent (repeating IACI or adding a short course of prednisone can be considered). +TNF2: replace anti-TNF agent with another an anti-TNF agent or with an anti IL-6 agent (repeating IACI or adding a short course of prednisone can be considered)

In the Step-up arm, less severe patients will receive IACI in all affected joints without systemic therapies. If the juvenile arthritis disease activity score-10 (JADAS10) state of minimal disease activity (MiDA) after 3 months or the JADAS10 inactive disease (ID) state at 6 months are not reached, treatment with MTX will be started preferably subcutaneously in a single weekly dose of 15 mg/m2 (max 20 mg) and IACI can be repeated. Then, if the states of JADAS MiDA after 3 months or JADAS ID at 6 months are not reached, treatment with an anti-TNF agent in a single weekly dose of 0.8 mg/kg (max 50 mg) subcutaneously will be started. More severe patients in Step-up arm will receive IACI(s) and MTX as a first step and then escalate therapy with the same pathway, adding an anti-TNF treatment as a second step and switching to a different biologic medication as a third step (Fig. 1).

**Fig. 2:**
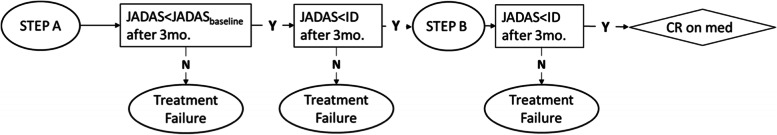
Step-down arm design. Step A is start methotrexate plus intra-articular joint injections for oligoarthritis without features of poor prognosis, methotrexate plus etanercept and optional intra-articular joint injections for other patients. Step B is withdraw methotrexate for oligoarthritis without features of poor prognosis, withdraw etanercept and continue methotrexate for other patients. JADAS: Juvenile Arthritis Disease Activity Score-10. JADAS<ID: JADAS10 score below inactive disease cutoff. CR on med.: clinical remission on medication

Patients allocated to the Step-down arm (Fig. 2) will receive an early combined treatment (MTX plus IACI for less severe oligoarthritis, MTX plus etanercept for severe oligoarthritis and polyarthritis). After 6 months, if JADAS10 ID is achieved, less severe patients will have MTX discontinued, whereas more severe patients will stop etanercept and continue MTX. Children in Step-down arm experiencing a worsening from the baseline of the JADAS10 at 3 months, not achieving JADAS10 ID at month 6, or losing the state of ID after month 6 will be considered as treatment failures and will change treatment according to the attending physician decision.

For the IACIs, triamcinolone hexacetonide will be administered at a dose of 1 mg/kg (max 40 mg) in the hips, knees and shoulders, at the dose of 0.75 mg/kg (max 30 mg) in the ankles and elbows, at the dose of 0.5 mg/kg (max 20 mg) in the wrists; methylprednisolone acetate will be administered at the dose of 1 mg/kg (max 40 mg) in the subtalar and inter-tarsal joints and at the dose of 5–10 mg, depending on the size of the child, in the small joints of hands and feet. A course of oral prednisone instead of or in addition to joint injection can be prescribed in case hip, temporomandibular joints or cervical spine are involved, or in case more than four joints are affected or joint injections cannot be performed.

### Patients’ assessment and disease activity monitoring

Complete physical examination with joints assessment, main patient/parent reported outcomes collection (including pain level, morning stiffness duration, disease activity self-assessment, physical function, and health-related quality of life), uveitis screening, medication history, treatment adverse events evaluation, and laboratory tests (haematological and biochemical analysis and urinalysis) will be performed at each trial visit.

The level and state of disease activity will be measured in all patients at each step of the study by means of the JADAS10. The JADAS10 [18] is a composite disease activity score validate for use in children with JIA and includes the following 4 measures: physician’s global assessment of disease activity, measured on a 0–10 21-circle Visual Analogue Scale (VAS) where 0 = no activity and 10 = maximum activity; parent/patient global assessment of well-being, measured on a 0–10 21-circle VAS where 0 = very well and 10 = very poor; the count of joints with active disease cut at 10 joints (i.e. 1 point for each active joint up to a maximum of 10 points); and the erythrocyte sedimentation rate (ESR), normalised to a 0 to 10 scale according to the following formula: (ESR (mm/hour) – 20)/10. Before making the calculation, ESR values < 20 mm/hour are converted to 20 and ESR values > 120 m/hour are converted to 120. The definition of the disease states of ID, MiDA, and high disease activity (HDA) based on the JADAS10 refers to the newest version of JADAS10 cut-off values [19, 20]. New cutoffs for oligoarthritis and polyarthritis are shown in Table 2. Data will be collected electronically in the PRINTO web-based platform designed for the PharmaChild registry.

**Table 2:** New JADAS10 and cJADAS10 cutoffs for disease activity states (Refs [16, 17])

	JADAS10	cJADAS10
**Oligoarthritis**		
Inactive disease	≤1.4	≤1.1
Minimal disease activity	1.5–4	1.2–4
Moderate disease activity	4.1–13	4.1–12
High disease activity	> 13	> 12
**Polyarthritis**		
Inactive disease	≤2.7	≤2.5
Minimal disease activity	2.7–6	2.5–5
Moderate disease activity	6.1–17	5.1–16
High disease activity	> 17	> 16

### Endpoints

The effectiveness of the two therapeutic strategies will be compared by assessing the frequency of clinical remission (CR) at 12 months [21]. CR is defined as the persistence of the JADAS10 state of ID for at least 6 months. Patients will be considered to be in ID from the day of the first visit with JADAS10 ID until the day before the first subsequent visit in which the patient is found to have lost the state of JADAS10 ID. Then, a number of secondary endpoints will be assessed in order to better understand the efficacy of the two treatment strategies (Table 3). The rate of patients achieving the JADAS-defined and the ACR-defined state of ID, the time to achieve ID and clinical remission, the time spent in inactive disease, the cumulative level of disease activity and the rate of uveitis will be compared between the two arms.

**Table 3:** STARS trial endpoints

**Primary endpoint**	
Clinical remission on or off medication at 12 months.	
The effectiveness of the two therapeutic strategies will be compared by assessing the frequency of clinical remission (CR) at 12 months. CR is defined as the persistence of the JADAS state of ID for at least 6 months	
**Secondary endpoints**	
Inactive disease	
The rate of patients who achieve the JADAS/JIA ACR state of ID at any single point in time throughout the study period will be compared between the 2 arms.	
Time to inactive disease as per JADAS/JIA ACR criteria	
Time to achieve the JADAS/JIA ACR state of ID will be calculated as the time difference (in days) between the date of randomization and the date of the visit on which the patient will be observed to be in ID.	
Time to JADAS/JIA ACR clinical remission	
Time to achieve the JADAS/JIA ACR state of clinical remission will be calculated as the time difference (in days) between the date of randomization and the date of the visit on which the patient will be observed to be in clinical remission (i.e. persistent inactive disease for at least 6 months).	
Time spent in JADAS/JIA ACR inactive disease	
The cumulative time spent in the JADAS/JIA ACR state of ID will be calculated as the time difference (in days) between the date of the first visit on which the patient will be observed to be in ID and the date on which he/she will be observed to be no longer in ID, that is when the disease will flare (see later for definitions), or database closure for analysis purposes. We will assume that if a patient is found to be in ID at 2 consecutive visits, the patient had ID on all days between these visits. If a patient will be found to have ID at a particular visit, but lost the ID status at the subsequent visit, the patient will be considered to have been in ID until the recurrence of active disease. Patients found to be in ID only at the time of database closure will contribute a single day of ID. The time in inactive disease per patient will be recorded and compared between the 2 arms.	
Cumulative level of disease activity throughout the study period	
The area under the curve of the JADAS10 score assessed at every study visit and the AUC of the parent version of the JADAS (parJADAS) assessed monthly will be recorded and compared between the 2 arms.	
Time spent on therapy	
The cumulative time on therapy will be calculated as the time difference (in days) between the date of the visit on which the patient will start a systemic medication (synthetic or biologic DMARDs or steroids) and the date on which he/she will be observed to no longer be in treatment with a systemic medication or completed the study. We assume that if a patient does not receive medications at 2 consecutive visits, the patient had not received medications everyday between these visits. Patients initiating a systemic treatment at the final visit of the study will contribute a single day of time in therapy. The mean percentage of time spent on therapy per patient will be recorded and compared between the 2 arms.	
Rate of flares	
The rate of patients who develop flare, defined as the recurrence of active disease after attaining inactive disease at last visit according to JADAS or JIA ACR definition, and the number of flares and the time to flare per patient will be recorded and compared. Notably, all patients prescribed intra-articular injections, synthetic or biologic DMARDs or systemic steroids will be considered as flare independently from JADAS or ACR criteria.	
Rate of uveitis onset	
The rate of patients who develop uveitis according to the Standardized Uveitis Nomenclature (SUN) will be recorded and compared between the 2 arms. The rate of patients requiring systemic medications for treatment of uveitis will be also recorded and compared between the 2 arms. However, these patients will be excluded from the study and followed for safety only.	

### Treatment adherence

Compliance will be monitored by study personnel at each study visit using diary cards and verbal information from the parent and/or subject. Reasons for missed doses must be recorded in the subject’s source documents and CRF.

Subjects provided with pre-filled syringes of investigational product will be instructed to return the empty packages and unused prefilled syringes in order to review drug accountability and subject compliance.

If more than four consecutive doses are missed due to adverse events (AEs), infections or surgeries, or if more than 2 consecutive doses are missed due to reasons other than AEs, infections or surgery, the Study Coordinating Centre must be consulted in order to determine whether the subject should continue taking investigational product.

### Sample size

Sample size calculations were extrapolated from a recent study of early aggressive therapy on JIA patients [11]. Using the sample size calculation for superiority trials with a delta of at least 20% (being the expected proportion of favourable outcome in the first arm p_1_ = 0.45 and in the second arm p_2_ = 0.25) and considering a drop out rate of 10%, a sample size of 109 patients in each group for a total of 218 patients will be required. Since an interim analysis is planned at enrolment of half of the sample, the total sample size was increased to 260 subjects. The trial will have 0.8 power for comparisons of the treatment arms, 0.05 type I error.

### Analysis plan

For proportional data the chi-square test or, where appropriate, the Fisher’s exact test will be applied. For continuous variables the t-test procedure or the ANOVA will be applied as appropriate. Non-parametric ANOVA will be applied in case of ordinal data or not-normally distributed variables; the Bonferroni correction will be applied for all a-posterior tests, in order to avoid multiple comparisons’ error. Treatment effect size will be calculated by dividing the difference between the baseline and the final visit value by the standard deviation of the first visit value. Survival analysis with censored data will be used in order to evaluate time to remission and time to flare. Survival curves will be drawn with the Kaplan-Meyer method and compared with the Log-Rank test. Emphasis will be placed on the intention-to-treat approach rather than on the analysis of the completers of the trial only. All analysis will be done in a blinded manner. Safety events are recoded by a medical monitor; all visit data are checked by the PRINTO coordinating centre and by a medical monitor. All moderate/severe AEs (including those leading to MTX or biologic treatment discontinuation) will be summarised per patient year of follow-up, describing the relationship to the treatment. The AE incidence rate by drug will be estimated after partitioning the follow-up periods of each patient into subintervals corresponding to the administration of the drug, with any event being attributed to the drug itself. This implicitly assumes the independence of the outcome in different subintervals pertaining to the same patient. Trial data will be entered online by the participating centres on a secure web system managed by the coordinating calculated to compare the primary AE rates with the remaining comparator groups. As possible covariate for the analysis JIA category, gender, age and drug use history will be considered.

### Trial registration

The Trial is registered on the ClinicalTrials.gov registry (NCT03728478) on October 31st, 2018 and EU Clinical Trials Register on May 14th, 2018, Eudract Number 2018–001931-27.

### Study organisation and funding

The STARS trial is coordinated from the Paediatric Rheumatology InterNational Trials Organisation (PRINTO at www.printo.it) coordinating centre in Genoa, Italy. It is an independent trial funded by the Italian Drug Agency (*Agenzia Italiana del Farmaco*) and the *Compagnia di San Paolo*. The trial has an Independent Data and Safety Monitoring Board. In Italian centres, Etanercept as a first-line treatment in subjects in the Step-down arm is provided by Pfizer through an Investigator Initiated Research grant.

## Discussion

This innovative trial compares the effectiveness of a conventional therapeutic regimen, based on treatment escalation and driven by the treat-to-target approach with that of an early aggressive combined intervention, including the first-line treatment with an anti-TNF agent in polyarthritis and more severe oligoarthritis. We chose to adopt a very robust primary endpoint by comparing in the two arms the frequency of patients who will achieve the end of the study in clinical remission, i.e., maintaining the status of inactive disease for at least 6 months. Therefore, if the trial will show a superiority of the more aggressive arm, we will provide important evidence supporting the theory of the existence for JIA patients of a window of opportunity, an early phase of the disease where treatment can possibly alter significantly the evolution of the disease. Furthermore, the comparison with an historical cohort of JIA children with the same disease features will allow us to demonstrate the effectiveness of two treatment strategies, both based on tight patient monitoring and treatment adaptation according to standardized disease activity measurement.

We acknowledge that the STARS trial has several potential limitations. One is the choice, for sake of uniformity, of etanercept as a first-line biologic medication in both arms. Etanercept is still the most widely adopted choice for non-systemic JIA and it is the biologic drug for which we have the longest experience on the field. However, we acknowledge that other drugs could also be the optimal choice in this population at higher risk for uveitis. Children requiring systemic medications due to uveitis will discontinue the trial and the comparison of the rate of uveitis between the two arms will be a secondary endpoint of the study.

### Trial status

**Fig. 3:**
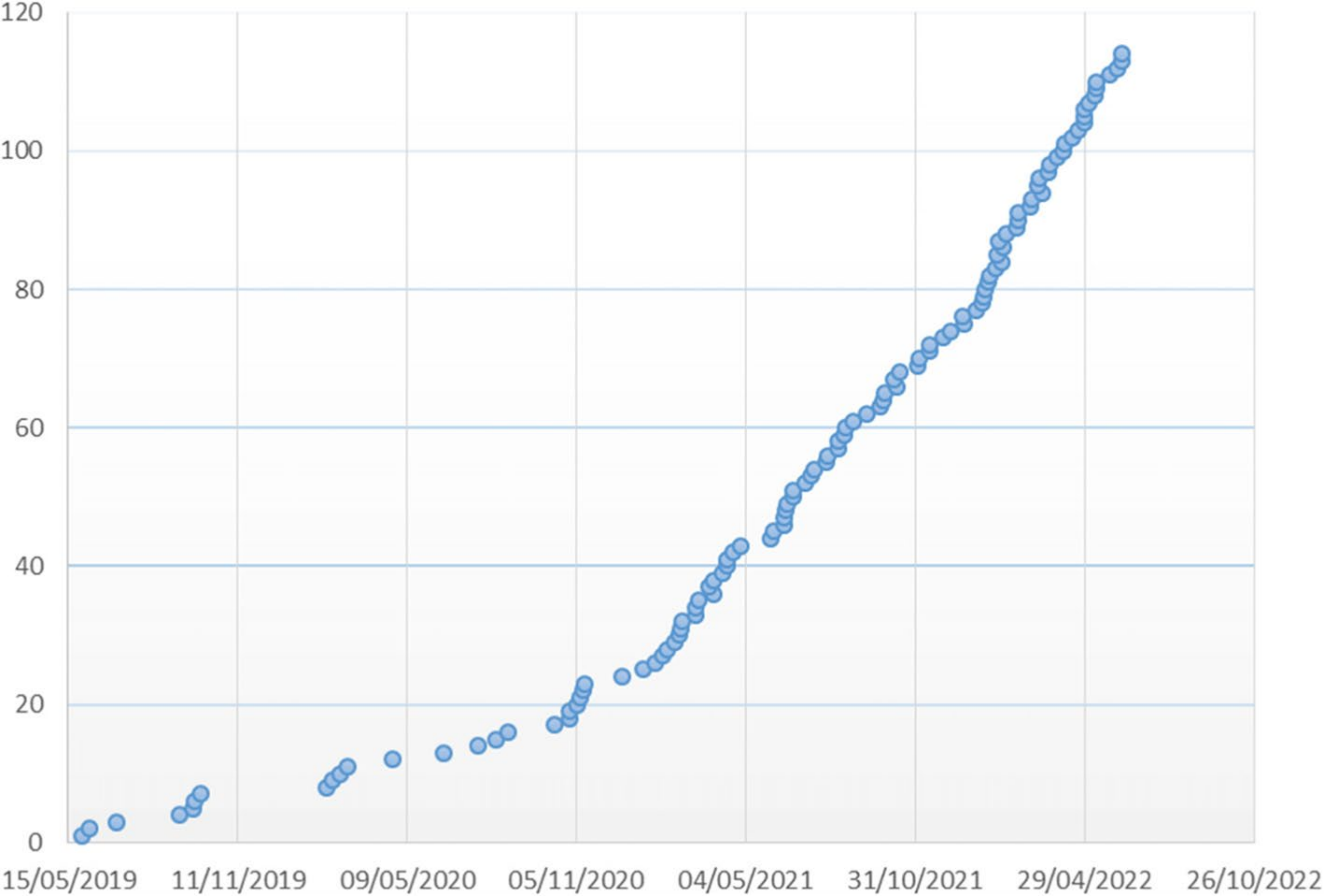
Trial enrolment progression. In February 2021 most participating Italian centres obtained local IRB approval and we started monthly virtual Town Hall Meetings

This summary is based on version 3.0 of the study protocol, dated 15th November 2021. At the time of submission this trial is open in 22 hospital sites in Italy and has recruited 113 participants (Fig. 3), 57 in the less severe group, 56 in the more severe group. Fifty-seven children were randomised in the Step-up arm, 56 were randomised in Step-down arm. In the whole sample, the number of subjects lost at follow-up was 2. In general, recruitment has revealed to be more difficult than expected. This was mainly due to the COVID19 pandemics and to delays in the activation of secondary centres, as well as to the issue of randomization being an important barrier to enrolment. Indeed, subjects in the trial are children with very recent onset JIA and their families are at the beginning of the journey with this disease: even after a detailed description of the risk/benefit ratio in both arms, accepting that the first-line treatment is decided by a machine was a tough choice.

## Data Availability

The datasets generated and/or analysed during the current study are available in the PRINTO repository (www.printo.it). The datasets used and analysed during the current study are available from the corresponding author on reasonable request.

## Abbreviations

AE: Adverse Event
CR: Clinical Remission
CRF: Case Report Form
DMARD: Disease Modifying Antirheumatic Drugs
ESR: Erythrocyte Sedimentation Rate
IACI: Intra-articular Corticosteroid Joint Injections
ID: Inactive Disease
JADAS10: Juvenile Arthritis Disease Activity Score-10
JIA: Juvenile Idiopathic Arthritis
MiDA: Minimal Disease Activity
MTX: Methotrexate
NSAID: Non-Steroidal Anti-Inflammatory Drugs
VAS: Visual Analogue Scale
